# Variation in the Aroma Composition of Jasmine Tea with Storage Duration

**DOI:** 10.3390/foods13162524

**Published:** 2024-08-13

**Authors:** Zihao Qi, Wenjing Huang, Qiuyan Liu, Jingming Ning

**Affiliations:** State Key Laboratory of Tea Plant Biology and Utilization, Anhui Agricultural University, Hefei 230036, China; qzh200411@163.com (Z.Q.); hwj5740@163.com (W.H.); lqy990913@163.com (Q.L.)

**Keywords:** jasmine tea, storage, GC-O, SBSE, aroma intensity, (*Z*)-4-heptenal

## Abstract

This study investigated the changes in the aroma of jasmine tea during storage. Solid-phase micro-extraction (SPME)–gas chromatography (GC)-mass spectrometry (MS) and stir bar sorptive extraction (SBSE)-GC-MS were combined to detect all volatile compounds. GC-olfactometry (GC-O), odor activity value (OAV), and *p*-value were employed to analyze and identify the key aroma compounds in six jasmine tea samples stored for different durations. Nine key aroma compounds were discovered, namely (*Z*)-3-hexen-1-yl acetate, methyl anthranilate, methyl salicylate, trans-*β*-ionone, linalool, geraniol, (*Z*)-4-heptenal, benzoic acid methyl ester, and benzoic acid ethyl ester. The importance of these compounds was confirmed through the aroma addition experiment. Correlation analysis showed that (*Z*)-4-heptenal might be the main reason for the increase in the stale aroma of jasmine tea. Through sensory evaluation and specific experimental analysis, it can be concluded that jasmine tea had the best aroma after 3 years of storage, and too long a storage time may cause the overall aroma of the tea to weaken and produce an undesirable odor. The findings can provide a reference for the change in aroma during the storage of jasmine tea and provide the best storage time (3 years) in terms of jasmine tea aroma.

## 1. Introduction

Jasmine tea is made by mixing fresh jasmine with tea [1]. It is popular due to its long-lasting aroma and smooth taste, and it is used as a medicinal herb to calm nerves [2] and relieve depression [3]. The total production of jasmine tea reached 114.1 kt in 2022, the gross product was reported to be 14 billion yuan, and its total sales volume accounted for 5.53% of China’s tea market [4]. The jasmine tea industry is ushering in broad market prospects because of its unique floral aroma and health effects. Consumers’ preferences for natural and healthy beverages and the rise of tea culture will provide a strong impetus for the further development of jasmine tea.

The storage of tea will influence the aroma composition and thus affect the tea’s quality. The traditional view is that there is a significant positive correlation between the aroma and quality of jasmine tea; therefore, aroma has become an important evaluation index. Over 70 aroma compounds have been identified in jasmine tea, and the content of aroma compounds accounts for the highest proportion of all teas [5]. Among them, volatile compounds such as methyl anthranilate, (*E*)-2-hexenyl hexanoate, linalool 4-caprolactone, and 4-hydroxy-2, 5-dimethyl -3(2H)-furanone are powerful odorants in jasmine tea [2]. Various studies have identified that the primary volatile compounds present in jasmine tea include (*Z*)-3-hexen-1-yl acetate, linalool, benzyl benzoate, α-farnesene, methyl salicylate, methanol benzene, (*Z*)-3-hexen-1-yl benzoate, methyl anthranilate, and indole. These compounds predominantly exist in jasmine as bonded glycosides and are released via endogenous mechanisms. Furthermore, it has been noted that the aroma of jasmine is liberated through enzymatic processes, with *β*-evening primrose and *β*-glucosidase being crucial contributors to this phenomenon [1]. Currently, research on jasmine tea has mainly focused on enhancing processing methods and extraction techniques [6], analyzing volatile compounds [3], and the effect of jasmine on aroma [7]. However, changes in the aroma of jasmine tea during storage have not been reported. Regarding the effects of storage on other tea types, fresh dark tea may have the disadvantage of an excessively smoky aroma [8], but some flavor compounds produced by microbial metabolism during storage may improve the aroma properties of tea [9]. Furthermore, freshly processed white tea has a grassy, tender corn aroma and a slightly thin aroma [10], but white tea has a strong, sweet aroma and a medicinal herb aroma after storage [11]. Taken together, the aroma and overall quality of tea can change considerably during storage. Therefore, it is crucial to understand the impact of storage duration on the aroma of jasmine tea for the preservation of jasmine tea and the provision of consumer choice.

The objective of this study was to investigate the variations in the aroma of jasmine tea with varying storage times. Traditional extraction methods for tea aroma include headspace solid phase micro-extraction (HS-SPME), solvent-assisted flavor evaporation (SAFE), stir bar sorptive extraction (SBSE), solid-phase extraction (SPE), simultaneous distillation extraction (SDE) and supercritical fluid extraction (SFE). Among all methods, HS-SPME demonstrates effective analysis of some small-molecule alcohols and terpenes at high concentrations [12], and SBSE has a good absorption effect on macro-molecular esters and nitrogen heterocycles [13]. Therefore, in order to extract as many volatile compounds as possible from tea infusion, we combined HS-SPME and SBSE to extract. Screening of key aroma compounds in jasmine tea was achieved through the integration of odor activity value (OAV), *p*-value, and gas chromatography-olfactometry (GC-O) technology. By analyzing the alterations of key aroma compounds, we could discover the changes in aroma components during the storage of jasmine tea. This can help develop the best storage strategy to maintain the aroma quality of jasmine tea.

## 2. Materials and Methods

### 2.1. Samples

Samples were produced by Fujian Min Rong Tea Co. Ltd., packaged completely and with specific labels, and stored in a ventilated, dry, and light-proof warehouse at a temperature of about 20 °C and humidity of about 50%. The jasmine tea sample was made by mixing silver needle white tea and jasmine flowers to absorb the aroma (throughout the production process, the weight ratio of jasmine and white tea used was 1:1), picking out the jasmine flowers after the absorption was completed, then added new jasmine and repeated these steps, after carrying out the above process three times, the jasmine tea was dried with a water content of 8.0–8.5%, and finally packed for storage. All the raw materials of the samples were from the same origin, picked in the same season, and prepared by the same processing technology. All tea samples were obtained in April 2023 and produced in 2022, 2021, 2020, 2019, 2018, and 2011. The samples were collected and placed in a laboratory freezer at −20 °C.

### 2.2. Chemicals and Materials

SPME fibers and a micro-extraction fiber head; PDMS twister for SBSE; gas chromatograph with a mass detector and an olfactory detection port (Agilent 7890B); an HP-5MS capillary column; a blue polytetrafluoroethylene (PTFE)/white silica gel spacer; 20-mL spiral vials; 250-mL conical flasks (Synthware Glass Instrument Co., Ltd., Beijing, China); an AB104-N electronic balance (Jinghong Experimental Equipment Co., Ltd., Shanghai, China); and a DF-101S constant-temperature magnetic stirring water bath (Yuhua Instrument Co., Ltd., Gongyi, China).

The materials used were sodium chloride (Sinopharm Chemical Reagent Co., Ltd., Shanghai, China), C6-C25 n-alkane mixed samples (Dr. Ehrenstorfer GmbH, Augsburg Germany), ethyl caprate (≥99.9%), anhydrous ethanol (Shanghai Hutian Laboratory Equipment Co., Ltd., Shanghai, China), geraniol, linalool, methyl anthranilate, linalool oxide, (*Z*)-3-hexen-1-yl acetate, methyl salicylate, trans-*β*-ionone, (*Z*)-4-heptenal, benzoic acid methyl ester, and benzoic acid ethyl ester (the concentrations of these standard compounds were >98%).

### 2.3. Extraction and Analysis of Volatile Compounds

#### 2.3.1. Extraction of Volatiles by HS-SPME

In a conical flask, 3 g of dried tea was added, and then 150 mL of boiling water was steeped for 5 min. Then, the tea infusion underwent filtration into a new conical flask, followed by cooling to ambient temperature. Subsequently, 10 mL of the tea infusion, 30 µL of internal standard solution (10 µL/mL aqueous ethyl caprate), and 3 g of NaCl were sequentially added to a 20-mL spiral HS bottle. After shaking well, the spiral HS bottle was placed in a 40 °C magnetic water bath stirrer and stirred for 15 min. Finally, the extracted fiber was pushed into the spiral HS bottle using an extraction needle and allowed to adsorb for 30 min [14].

#### 2.3.2. Extraction of Volatiles by SBSE

The experimental steps are described in Section 2.3.1. SBSE extraction differs in that it involves the use of a rotor (PDMS twister) to absorb volatile compounds with an extraction duration of 90 min [15].

#### 2.3.3. Gas Chromatograph-Mass Spectrometry (GC-MS) Separation and Identification of Volatile Compounds

A gas chromatograph (Agilent 7890B) with a mass detector in combination with an HP-5MS capillary column can be used to separate and identify a wide range of volatile compounds. After extraction, the SPME fiber was inserted into the inlet for thermal desorption for 5 min at 250 °C and then pulled out. The PDMS twister was inserted into a thermal desorption unit (TDU, Gerstel, Mülheim an der Ruhr, Germany). The TDU desorption program involved maintaining the temperature at 30 °C for 1 min, followed by an increase to 240 °C (maintained for 5 min) at a rate of 100 °C/min. The cooled injection system 4 (CIS 4) was initially kept at −100 °C using 99.999% liquid N_2_, and then raised to 280 °C (maintained for 3 min) at a rate of 12 °C/s from −100 °C (maintained for 1 min) after desorption. The GC oven temperature was programmed as follows: for HS-SPME, the column chamber was heated to 40 °C and kept for 3 min, and subsequently gradually raised to 160 °C at a rate of 4 °C/min. Then increased to 250 °C at a speed of 12 °C/min. The temperature remained constant for 5 min, and the oven heating procedure of SBSE was the same as that of HS-SPME. A mass-selective detector was used in the positive-ion mode, with a mass scan conducted in the range of 30–350 *m*/*z* and a scannable energy of 70 eV. The actual retention index (RI) was calculated based on the time of detection of the compounds and the retention time of the n-alkanes mixture (C6–C25), and then compared to the RI* in the NIST 2020 library, the difference within ±20 was considered to be a volatile compound. The concentration of volatile compounds was relatively quantified by comparing the peak area of the compound with the peak area of the added ethyl caprate (Table S1).

#### 2.3.4. Analysis of Key Aroma Compounds by GC-O and Aroma Intensity (AI)

The GC-O uses the Agilent 7890B GC with an olfactory detection port and mass detector. The analysis column’s gas outlet was divided into two identical sections, with half of the gas flowing to the MS detector (250 °C) and the other half flowing to the olfactory detection port (230 °C). The carrier gas, which was 99.999% pure helium, flowed at a linear velocity of 40 cm/s. The HS-SPME and SBSE methods and their heating procedures were consistent with those described in Section 2.3.1, Section 2.3.2 and Section 2.3.3. The sniffing team consisted of three laboratory personnel with specialized sensory training who were not allowed to sniff continuously. They recorded the aroma characteristics through an olfactory detection port and rated the intensity of the odor from 0 to 4 (0–1, very weak; 1–2, weak; 2–3, strong; 3–4, very strong). The sniffing results recorded by the three sniffers were summarized, and if they recorded different aroma characteristics, sniffing was repeated [16].

### 2.4. Quantitative of Key Aroma Compounds and Calculation of OAV

In order to screen key aroma compounds, we employed a standard addition method combined with selected ions for quantification. Briefly, a 1000 μg/mL stock solution of reference compounds was prepared, and a certain concentration of reference compounds was added to the tea infusion. HS-SPME and GC-MS were operated under identical conditions, as outlined in Section 2.3. Calibration curves were created using six target concentrations that varied from 0.01 to 100 μg/L. Quantitative ion integration was performed using the collected reference odorants. In the calibration curves, the original peak area of the sample was subtracted from the peak area of the target reference odorant as the ordinate of the calibration curve, and the concentration of the added reference odorant was the horizontal coordinate. Information on the calibration curves is presented in Table S3.

The ratio of a single volatile compound’s concentration in a sample to its odor threshold was known as the OAV. This parameter’s calculation algorithms refer to earlier research and can be used as a gauge of the compound’s odor potency [17].

### 2.5. Quantitative Descriptive Analysis (QDA) and Aroma Addition Experiment

QDA was employed to analyze the characteristics and intensity of the aroma of the tea infusion samples [18]. Tea sniffers (6 men and 6 women, 20–30 years old) with considerable experience in sensory evaluation sniffed the jasmine tea infusion and indicated six aroma terms that fit the characteristics of jasmine tea, including stale, coconut-like, fruity, floral, fresh, or sweet. The six samples were evaluated blindly without the tea sniffing team knowing the details of the samples. The tea sniffing team employed a 5-point scoring system (0–1, very weak; 1–2, weak; 2–3, medium; 3–4, strong; 4–5, very strong) to reflect the strength of the aroma [19].

An aroma addition experiment was conducted to investigate the influence of the screened key aroma compounds on the tea infusion. Compounds with AI > 2, *p* < 0.05, and OAV > 1 were selected as key aroma compounds, and the highest concentrations of these key aroma compounds in six samples were used as standard concentrations and the difference between each key aroma compound and its standard concentration in each sample was calculated. According to the calculated concentration difference, compounds with corresponding concentrations were added to the six tea infusions so that the concentration of each key aroma compound in the six tea infusions was the same, which means that these key aroma compounds all reached the standard concentration. Finally, QDA experiments were performed on these tea infusions to further determine whether the selected key aroma compounds were correct [20].

The tea infusion samples were prepared according to the GB/T 23776-2018 standard [21]. First, we accurately weighed 3 g of tea samples and brewed them with 150 mL of boiling water for 5 min [7], then filtered and placed them into sniffing bottles. Finally, the sniffing bottle was transferred to a constant temperature water bath at 50 °C, waiting for sniffing.

### 2.6. Statistical Analysis

Multivariate statistical analyses were performed utilizing SIMCA-P (version 14.1, Umetrics, Umea, Sweeden), and significant differences in volatile compounds were calculated using SPSS (version 26; IBM, Armonk, NY, USA), *p* < 0.05 was considered to represent statistical significance. Graphs were plotted using Origin software. All data were visualized using Origin 2023b, Cytoscape v3.7.2 (OriginLab Corp., Northampton, MA, USA), and TBtools-II v2.012. The experiments were repeated thrice, and the average result is shown with the ± standard deviation.

## 3. Results and Discussion

### 3.1. Traditional Sensory Evaluation of Jasmine Tea Stored for Various Durations

Sensory evaluation has always been considered the most intuitive way to judge the quality of tea [22]. It uses vision, smell, taste, touch, and other sensory organs to identify the taste of tea, aroma, shape, and color to determine tea quality. The results of the aroma sensory evaluation are displayed in Table 1. By sniffing, it could be clearly felt that the 2011 sample had a stale aroma, and the 2020 sample had a better aroma than the other samples; the fruity and floral aroma was better, and it lasted longer.

### 3.2. Quantitative Analysis of Total Volatile Compounds in Jasmine Tea

**Figure 1:**
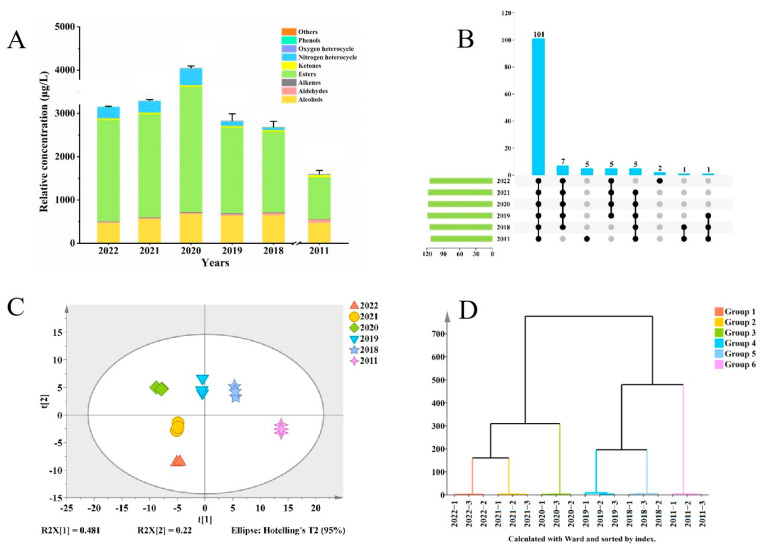
Variations in the volatile components of jasmine tea throughout storage. (**A**) Relative concentrations of overall and several classes of volatile compounds. (**B**) Volatiles shared and not shared among the six samples. (**C**) Principal component analysis (PCA). (**D**) Hierarchical clustering analysis (HCA).

A total of 127 volatile compounds were detected by HS-SPME and SBSE combined with GC-MS: 52 esters, 24 alcohols, 14 terpenes, 13 aldehydes, 11 ketones, three phenols, two nitrogen heterocycles, two oxygen heterocycles, and six other compounds (Table S1). Figure 1B shows that 101 of 127 volatile compounds were present in all samples of jasmine tea. Five of these compounds could only be detected in the 2011 sample: pentanal, 2-ethyl-furan, butanoic acid methyl ester, 3-methyl-1-butanol, and benzeneacetaldehyde. Figure 1A shows that esters had the highest proportion of these aromatic compounds, followed by alcohols and aldehydes, as reported in previous studies [1,7]. The total concentration of these three types of compounds accounted for 91.72–98.06% of the overall volatile compound concentration (Table S1). The concentrations of most of the volatile compounds exhibited an initial rise followed by a subsequent decline as the storage period extended. For example, the ester concentration was 2346.467 μg/L in the 2022 sample, increased to 2897.34 μg/L in the 2020 sample, and decreased to 973.469 μg/L in the 2011 sample. In contrast, the concentration of aldehydes increases with an increase in storage time, being 64.853 μg/L in the 2011 sample but only 16.614 μg/L in the 2022 sample, but most aldehydes may bring a fatty aroma to jasmine tea [23]. Notably, the concentration of total volatile compounds was the highest in the 2020 sample, being 4050.985 μg/L, and the floral, fruity, and fresh characteristics of the jasmine tea were the strongest in this sample. The 2011 sample with the longest storage time had the lowest aroma compound concentration, namely 1599.412 μg/L, and this concentration was significantly lower than that of the other five samples.

To investigate the changes in the characteristics of the volatile compounds of the jasmine tea samples during storage, concentration data for the 127 volatile compounds were subjected to Principal component analysis (PCA) and Hierarchical clustering analysis (HCA) as shown in Figure 1C and D [24]. The PCA results showed differences between samples stored for different times. Especially the sample stored in 2011 showed significant separation from the other samples, which might be due to the significant changes in the volatile compounds caused by the long storage time. In addition, there was good data variance for the first two principal components, which explained 48.1% and 22% of the population variation, respectively. As shown in Figure 1D, the samples were able to be clearly classified into four categories: 2022 and 2021 formed one category; 2019 and 2018 formed another; and 2020 and 2011 represented two separate categories. From this, we speculate that jasmine tea samples will reach a turning point in 2020, when the volatile compounds show significant changes, leading to a change in the aroma of jasmine tea.

### 3.3. Changes in the Concentration of Volatile Compounds during Storage

**Figure 2:**
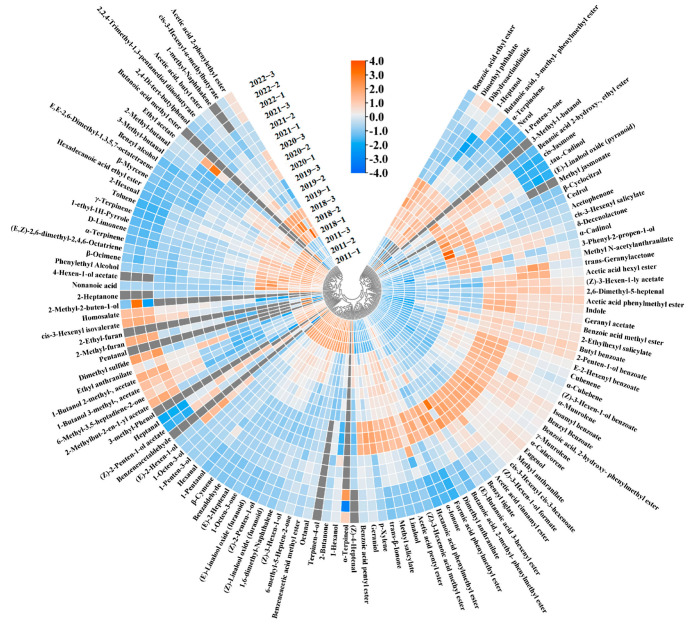
Heat map of all volatiles in six jasmine tea samples. Gray represents volatiles that were not detected by gas chromatograph-mass spectrometry (GC-MS) in the samples.

Quantitative findings from earlier experiments have indicated a noticeable shift in the concentration of numerous volatile compounds over the course of storage. To visualize the concentration variations of the volatile compounds in the jasmine tea samples stored for different durations, we conducted a heat map analysis. The data presented in Figure 2 illustrate that the levels of the most volatile compounds in jasmine tea rose at the beginning of the storage period, followed by a gradual decline in the later stages of storage; this change is consistent with previous research [25]. Moreover, changes in the levels of volatile compounds could be broadly classified into three categories. These changing patterns in the concentrations of volatile compounds during storage also occurred in the previous storage of Pu-erh tea [25] and large-leaf black tea [26]. In the first category, the concentration increased and then decreased with storage time; in the second category, it increased with storage time; and in the third category, it decreased with storage time. About 50% of the 127 volatile compounds belonged to the first category, and the primary aromas of the overall volatile compounds were floral and fruity, most of which were highest in the 2020 sample. Notably, the ester concentration, which is beneficial to the aroma of jasmine tea, first increased from 2346.467 μg/L in the 2022 sample to 2897.34 μg/L in the 2020 sample and then decreased to 973.469 μg/L in the 2011 sample. In addition, esters with fruity aroma (e.g., acetic acid pentyl ester, benzoic acid ethyl ester, and benzoic acid methyl ester), lactones with sweet aroma (e.g., *δ*-decenolactone and dihydroactinidiolide), ketones with floral aroma (e.g., trans-*β*-ionone), and anthranilates with grape aroma (e.g., methyl anthranilate), which is unique to jasmine tea [5], the change in these compounds was consistent with the above-mentioned ester total concentration, and the concentrations were the highest in the 2020 sample. The second category of volatile compounds mainly includes aldehydes with almond and malt aromas (e.g., benzaldehyde, 2-methylbutyraldehyde, and 3-methylbutyraldehyde) and alkenes with citrus aroma (e.g., *D*-limonene, *β*-ocimene, and *β*-myrcene). The third category is mainly composed of small molecules and esters with grass aroma (e.g., cis 3-hexenyl isovalerate), which indicates that the grass aroma of jasmine tea gradually decreases with storage.

### 3.4. GC–O Screening of Key Aroma Compounds

In order to assess the influence of diverse volatile compounds present in jasmine tea on its overall aroma, we separated all volatile compounds using GC-O. Subsequently, we identified aroma-active compounds that could potentially enhance the aroma of jasmine tea by conducting a sniffing evaluation. Three individuals with professional aroma training smelled the volatile compounds, which were separated from six jasmine tea samples, and they identified 37 aroma compounds with aroma activity (Table 2), including 14 esters, nine alcohols, six aldehydes, three alkenes, three ketones, one nitrogen heterocycle, and one benzene compound. Most of the 37 aroma compounds discovered in the olfactory detection had floral or fruity aroma.

Of the 37 compounds detected through GC-O, most esters had floral and fruity aromas. Except for methyl salicylate, which has a mint aroma. Most of the alcohols also had a fruity and floral aroma. These findings correspond with the typical aroma characteristics of jasmine tea. After the preliminary screening, 11 aroma compounds with AI > 2 were found in the six samples, including linalool, methyl salicylate, methyl anthranilate, benzoic acid methyl ester, (*Z*)-3-hexen-1-yl acetate, geraniol, trans-*β*-ionone, (*E*)-linalool oxide (furanoid), benzoic acid ethyl ester, (*E*)-linalool oxide (pyranoid), and(*Z*)-4-heptenal. The 11 compounds identified were provisionally regarded as the key aroma compounds involved in the storage progress of jasmine tea.

### 3.5. Identification of Key Aroma Compounds in Jasmine Tea

Variations in the levels of key aroma compounds varied significantly among the six samples with varying storage duration (*p* < 0.05; Table 2), and the AI and *p*-values could be used together to screen the key aroma compounds [18]. Because the AI in GC-O is subjective, it is difficult to establish a standard. Therefore, we also examined the OAV (absolute concentration/threshold) of the 11 key aroma compounds. The OAV is a measure of the extent to which a compound contributes to the overall aroma of a sample. The internal standard method was utilized to determine the absolute concentrations of the 11 key aroma compounds across a concentration range of 0.01–100 μg/L. Data were obtained by adding different concentrations of standards to the tea infusion and then analyzing the infusion using GC-MS. Standard curves were utilized to determine the concentration of the 11 primary aroma compounds in the tea infusion (Table S3), followed by the computation of the respective OAV values. Compounds are typically deemed detectable by smell only if their OAV > 1 [27]. Based on the OAV, nine key aroma compounds were screened (Table 3), including (*Z*)-4-heptenal, (*Z*)-3-hexen-1-yl acetate, benzoic acid methyl ester, methyl salicylate, methyl anthranilate, trans-*β*-ionone, linalool, benzoic acid ethyl ester, and geraniol. The majority of key aroma compounds exhibit floral and fruity aromas; among them, linalool, trans-*β*-ionone, geraniol, and methyl anthranilate are generally considered the main contributors to floral and fruity aromas in tea [1,18]. With the exception of (*Z*)-4-heptenal, which had a stale and fish oil aroma, methyl salicylate had a mint aroma. The concentrations of eight key aroma compounds first increased and then decreased, among which six key aroma compounds exhibited the highest OAV for the 2020 sample, including methyl salicylate (OAV = 11.6), methyl anthranilate (OAV = 111.2), (*Z*)-3-hexen-1-yl acetate (OAV = 17.3), trans-*β*-ionone (OAV = 521.7), linalool (OAV = 464.9), and geraniol (OAV = 7.2). All six compounds exhibited floral and fruity aromas. The highest OAV of benzoic acid methyl ester (OAV = 8.2) was obtained for the 2021 sample, and the highest OAV of benzoic acid ethyl ester (OAV = 3.3) was obtained for the 2019 sample. Whereas the OAV for (*Z*)-4-heptenal was the highest in the 2011 sample (OAV = 418.3). The correlation analysis findings indicated a strong positive correlation between (*Z*)-4-heptenal and the duration of storage (Table S2). Therefore, (*Z*)-4-heptenal of a fish oil aroma might be the main compound leading to jasmine tea having a stale aroma. In summary, the concentration of key aroma compounds first increased and then decreased during storage, and floral and fruity aromas dominated in the 2020 sample, while the stale aroma dominated in the 2011 samples. This finding was consistent with the sensory evaluation of the aroma quality of different samples.

### 3.6. Quantitative Descriptive Analysis

**Figure 3:**
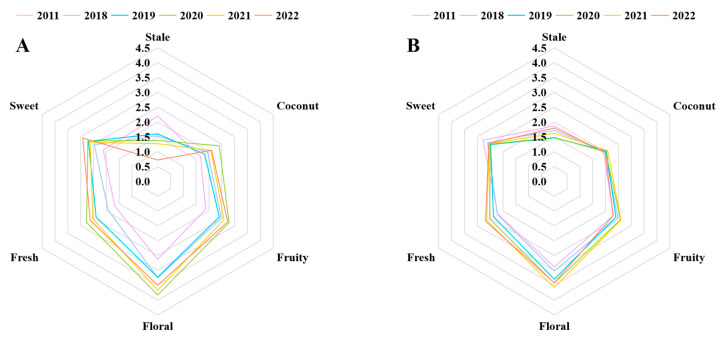
(**A**) Quantitative descriptive analysis (QDA) radar map of six sample infusions. (**B**) QDA radar map of six sample infusions after adding nine key aroma compound standard solutions.

To more clearly determine the differences between the six jasmine tea samples with different storage durations, we conducted a QDA experiment for aroma. The evaluation team provided scores for each jasmine tea infusion for the six aroma terms: stale, coconut, fruity, floral, fresh, and sweet [28]. The radar chart shows the fluctuation in the intensity of the fruity, floral, coconut, and fresh aromas during storage, which first increased and then decreased, and the highest intensity of the four aromas was in 2020. Whereas the intensities of the sweet and stale aromas decreased and increased, respectively, during storage (Figure 3A). Hence, it can be found that the change of six aromas and the concentration of corresponding aroma compounds was consistent.

To confirm whether the key aroma compounds jointly screened through GC-O, *p*-value, and OAV analysis significantly affected the aroma of jasmine tea, we conducted an aroma addition experiment [29]. After incorporating the key aroma compounds into the tea infusion at the appropriate concentrations, the evaluation team conducted an experiment using QDA [30]. Figure 3B indicates that the differences in the six aromas among the six samples were significantly reduced after adding the aroma compounds, which confirmed that the nine screened compounds were the key aroma compounds of jasmine tea [31]. Among the nine compounds, (*Z*)-4-heptenal was the only compound that was unfavorable to the aroma of jasmine tea and exhibited an increasing trend with storage time. Additionally, stale aroma was the only aroma that increased with storage duration. Therefore, this result further supports our speculation that (*Z*)-4-heptenal may be the primary reason for the gradual increase in the stale aroma of jasmine tea during storage. This finding is consistent with previous studies that indicated excessive storage could lead to elevated aldehyde levels, ultimately compromising the quality of the product’s aroma [23].

### 3.7. Metabolism of Key Aroma Compounds

Variations in the levels of key aroma compounds throughout storage can have a substantial impact on the overall aroma of jasmine tea. Linalool, geraniol, and methyl salicylate are all volatile monoterpenes, which originate from the breakdown of glycosides through hydrolysis during tea production and are important components affecting the aroma of jasmine tea [32]. Terpenoids are metabolically transformed from two C5 precursors, namely dimethylallyl diphosphate (DMAPP) and isopentenyl diphosphate (IPP). These two fundamental precursors are synthesized by the methylerythritol phosphate (MEP) pathway and mevalonate acid (MVA) pathway. The MEP pathway is involved in the production of monoterpenes, hemiterpenes, diterpenes, and volatile carotenoid derivatives, while the MVA pathway is responsible for the biosynthesis of sesquiterpenes, atypical terpenes, and geranyl terpenes. In some cases, carotenoid pigments may undergo cleavage by dioxygenase enzymes, resulting in the formation of volatile compounds like *α* and *β* ionones. The increase in trans-*β*-ionone levels at the beginning of storage may be due to the long-term autooxidation of carotenoids. Moreover, previous work has shown that the type and concentration of volatile autoxidation products of carotenoids increase when tea is stored at 20 °C [32,33,34]. Although methyl anthranilate, benzoic acid methyl ester, and methyl salicylate are highly similar in structure, their metabolic pathways involve completely different enzyme mechanisms, including multiple hydrolysis catalytic reactions [35]. (*Z*)-4-heptenal is the product of fatty acid oxidation. During tea storage, the oxidation of fatty acids is easily affected by the storage environment, especially the water content. Water content that is too high or too low can accelerate the oxidative degradation of fatty acids. Therefore, changes in the concentration of (*Z*)-4-heptenal over time during storage may be linked to variations in the moisture content of tea [36]. Taken together, the changes in the aroma of jasmine tea during storage may be attributed to natural oxidation, enzyme catalysis, and fluctuations in the storage environment.

## 4. Conclusions

In this study, we extracted 127 volatile components from six types of jasmine tea samples by HS-SPME and SBSE combined with GC-MS. Nine key aroma compounds were screened: (*Z*)-4-heptenal, (*Z*)-3-hexen-1-yl acetate, benzoic acid methyl ester, methyl salicylate, methyl anthranilate, trans-*β*-ionone, linalool, benzoic acid ethyl ester, and geraniol. Except for (*Z*)-4-heptenal, all other eight compounds contributed to the floral and fruity aromas of jasmine tea. The concentration of (*Z*)-4-heptenal with fish oil aroma showed an upward trend during storage, which may be the key reason for the increase in the stale aroma of jasmine tea during storage. It can be concluded that jasmine tea in 2020 (stored for 3 years) had the best aroma, and too long a storage time may cause the overall aroma of the tea to weaken and produce an undesirable odor. Our research results provide a new approach to improving the storage quality of jasmine tea by studying the important metabolic pathways affecting key aroma compounds.

## Figures and Tables

**Table 1:** Sensory experiments analyzed the aroma types of six jasmine tea samples.

Year	2022	2021	2020	2019	2018	2011
tea aroma	fresh and long-lasting	fresh, strong, and long-lasting	fresher, stronger, and longer-lasting	fresh, slightly strong, and lasting	fresh, slightly	stale aroma

**Table 2:** Gas chromatography-olfactometry (GC-O) analysis of all aroma compounds detected in the six samples through smell, and the aroma intensities (AI) and *p*-values of various compounds.

NO.	RI	Compounds	CAS	AI	Description	*p*
1	<700	2-Methyl-butanal	96-17-3	0.7	malty	<0.001
2	769	(*Z*)-2-Penten-1-ol	1576-95-0	1.8	musty	<0.001
3	797	Hexanal	66-25-1	1.7	green	<0.001
4	815	Acetic acid butyl ester	123-86-4	1.5	fruity	<0.001
5	855	(*Z*)-3-Hexen-1-ol	928-96-1	0.9	green	<0.001
6	868	p-Xylene	106-42-3	1.1	almond	<0.001
7	899	(*Z*)-4-Heptenal	6728-31-0	2.4	fish oil	<0.001
8	914	Acetic acid pentyl ester	628-63-7	0.9	fruity	<0.001
9	957	(*E*)-2-Heptenal	18829-55-5	0.7	rubble	<0.001
10	980	1-Octen-3-ol	3391-86-4	1.9	mushroom	<0.001
11	991	*β*-Myrcene	123-35-3	1.6	citrus	<0.001
12	1003	Octanal	124-13-0	0.9	citrus	<0.001
13	1008	(*Z*)-3-hexen-1-yl acetate	3681-71-8	2.3	fruity	<0.001
14	1047	Benzeneacetaldehyde	122-78-1	0.8	rose, honey	<0.001
15	1049	*β*-Ocimene	13877-91-3	1.9	citrus	<0.001
16	1076	(*Z*)-Linalool oxide (furanoid)	5989-33-3	1.9	citrus, floral	<0.001
17	1084	Formic acid phenylmethyl ester	104-57-4	1.5	fruity	<0.001
18	1095	(*E*)-Linalool oxide (furanoid)	34995-77-2	2.6	citrus	<0.001
19	1102	Benzoic acid methyl ester	93-58-3	2.6	fruity, sweet	<0.001
20	1115	Linalool	78-70-6	3.0	citrus	<0.001
21	1123	Phenylethyl Alcohol	60-12-8	1.2	rose, honey	<0.001
22	1134	*E*, *E*-2,6-Dimethyl-1,3,5,7-octatetraene	460-01-5	1.7	citrus	<0.001
23	1181	Benzoic acid ethyl ester	93-89-0	3.3	fruity	<0.001
24	1185	(*E*)-Linalool oxide (pyranoid)	39028-58-5	2.3	earthy	<0.001
25	1188	Benzeneacetic acid methyl ester	101-41-7	1.6	floral, fruity	<0.001
26	1208	Methyl salicylate	119-36-8	2.0	mint	<0.001
27	1257	Geraniol	106-24-1	3.0	citrus	<0.001
28	1262	Acetic acid 2-phenylethyl ester	103-45-7	1.1	honey, floral	<0.001
29	1306	Indole	120-72-9	1.3	camphor	<0.001
30	1356	Methyl anthranilate	134-20-3	2.2	grape, sweet	<0.001
31	1385	Geranyl acetate	105-87-3	1.9	floral	<0.001
32	1393	Butanoic acid 2-methyl phenylmethyl ester	56423-40-6	0.4	grape	0.003
33	1438	*α*-Ionone	127-41-3	0.8	violet-like	<0.001
34	1456	trans-Geranylacetone	3796-70-1	0.9	floral	<0.001
35	1483	Benzoic acid pentyl ester	2049-96-9	0.8	fruity, sweet	<0.001
36	1495	trans-*β*-Ionone	79-77-6	2.3	violet	<0.001
37	1505	Benzyl tiglate	37526-88-8	0.9	fruity	<0.001

**Table 3:** The odor activity value (OAV) of key aroma compounds in the six samples.

Compounds	Odor Quality	OT (μg/L in Water)	OAV					
			2022	2021	2020	2019	2018	2011
(*Z*)-4-Heptenal	fish oil	0.0087	—	154.8	19.2	117.8	375.6	418.3
(*Z*)-3-Hexen-1-ly acetate	fruity	8	13	15.2	17.3	5	10.8	5.3
Benzoic acid methyl ester	fruity	73	7.9	8.2	7.8	6.4	7.4	2.9
Methyl salicylate	mint	40	6.3	8.5	11.6	9.4	8.7	5.2
Methyl anthranilate	grape	3	78.9	80	111.2	58.9	56.7	45.6
trans-*β*-Ionone	floral	0.021	505.3	512.3	521.7	515.8	515	504.1
Linalool	citrus	0.6	262.8	345.9	464.9	405.2	422	220.6
Benzoic acid ethyl ester	fruity	53	0.39	0.6	0.2	3.3	2.6	0.9
Geraniol	citrus	1.1	4.7	4.8	7.2	4.8	4.7	3

## Data Availability

The original contributions presented in the study are included in the article/Supplementary Material, further inquiries can be directed to the corresponding author.

## Supplementary Materials

The following supporting information can be downloaded at: https://www.mdpi.com/article/10.3390/foods13162524/s1, Table S1: All detected volatiles in jasmine tea storage. Table S2: Correlation analysis between the concentration of 9 key aroma compounds and year of storage. Table S3: Standard curves of the concentrations of 9 key aroma compounds. Table S4: Reference odourants addition to the six tea infusions.
