# Supplementary material for: Variation in the Aroma Composition of Jasmine Tea with Storage Duration
Source: Foods. 2024 Aug 13;13(16):2524. doi: 10.3390/foods13162524 (PMC11353297; doi:10.3390/foods13162524)
Supplement: Supplementary file 1 [file foods-13-02524-s001.zip › foods-3122950-supplementary.pdf]

Supplementary files

Table S1
All detected volatiles in jasmine tea storage.

No	RI (HP-5MS)	Compounds	CAS	Class	Relative contents (μg/L)						Odor quality	Identification
					2022	2021	2020	2019	2018	2011		
1	<700	Dimethyl sulfide	75-18-3	others	1.36E-01	4.33E-02	4.34E-02	2.94E-02	Nd	Nd	putrid	MS, RI
2	<700	2-Butanone	78-93-3	ketones	3.35E-01	2.77E-01	4.25E-01	1.92E-01	3.43E-01	8.69E-01	ethereal, fruity	MS, RI
3	<700	2-Methyl-furan	930-27-8	oxygen heterocycle	3.23E-01	1.50E-01	1.68E-01	4.44E-02	Nd	Nd	——	MS, RI
4	<700	Ethyl acetate	141-78-6	esters	1.55E-01	3.11E-01	1.09E-01	1.08E-01	Nd	Nd	solvent-like	MS, RI
5	<700	3-Methyl-butanal	590-86-3	aldehydes	1.74E-01	1.11E-01	1.74E-01	2.84E-01	2.48E-01	4.20E-01	malty	MS, RI
6	<700	2-Methyl-butanal	96-17-3	aldehydes	2.49E-01	1.16E-01	1.80E-01	3.83E-01	3.30E-01	5.15E-01	malty	MS, RI,O
7	<700	1-Penten-3-ol	616-25-1	alcohols	1.72E-01	2.94E-01	2.34E-01	3.55E-01	7.74E-01	1.92E+00	pungent, milk-like	MS, RI
8	<700	1-Penten-3-one	1629-58-9	ketones	1.13E-01	1.82E-01	1.83E-01	2.81E-01	2.80E-01	Nd	pungent, train oil-like	MS, RI
9	<700	Pentanal	110-62-3	aldehydes	Nd	Nd	Nd	Nd	Nd	3.76E-01	green, fatty, moldy	MS, RI
10	<700	2-Ethyl-furan	3208-16-0	oxygen heterocycle	Nd	Nd	Nd	Nd	Nd	4.53E-01	——	MS, RI
11	714	Butanoic acid methyl ester	623-42-7	esters	Nd	Nd	Nd	Nd	Nd	7.46E-02	fruity	MS, RI
12	729	3-Methyl-1-butanol	123-51-3	alcohols	Nd	Nd	Nd	Nd	Nd	1.59E-02	malty	MS, RI
13	760	Toluene	108-88-3	others	5.37E-02	1.47E-01	2.21E-01	2.13E-01	2.25E-01	3.52E-01	chemical-like	MS, RI
14	765	1-Pentanol	71-41-0	alcohols	4.15E-02	9.63E-02	9.48E-02	8.81E-02	1.27E-01	2.35E-01	fruity, ethereal	MS, RI
15	769	(*Z*)-2-Penten-1-ol	1576-95-0	alcohols	6.24E-02	1.27E-01	1.06E-01	2.13E-01	2.37E-01	4.74E-01	musty	MS, RI,O
16	771	2-Methyl-2-buten-1-ol	4675-87-0	alcohols	2.51E-02	Nd	Nd	Nd	Nd	Nd	——	MS, RI
17	797	Hexanal	66-25-1	aldehydes	1.41E-01	1.83E-01	2.11E-01	2.38E-01	2.77E-01	8.28E-01	green	MS, RI,O
18	812	1-ethyl-1H-Pyrrole	617-92-5	nitrogen heterocycle	3.10E-02	9.33E-02	9.22E-02	9.40E-02	2.00E-01	2.01E-01	——	MS, RI
19	815	Acetic acid butyl ester	123-86-4	esters	9.25E-02	8.66E-02	9.70E-02	7.23E-02	1.87E-01	5.58E-02	fruity	MS, RI,O
20	853	2-Hexenal	505-57-7	aldehydes	2.63E-02	8.49E-02	8.49E-02	9.69E-02	1.35E-01	2.14E-01	——	MS, RI
21	855	(*Z*)-3-Hexen-1-ol	928-96-1	alcohols	9.07E+00	9.22E+00	8.19E+00	8.45E+00	1.21E+01	1.49E+01	green	MS, RI,O
22	867	(*E*)-2-Hexen-1-ol	928-95-0	alcohols	6.25E-02	1.20E-01	9.76E-02	6.14E-02	5.60E-02	Nd	fruity, ethereal	MS, RI
23	868	*p*-Xylene	106-42-3	others	1.42E-01	1.20E-01	3.90E-01	2.07E-01	2.33E-01	1.11E-01	almond	MS, RI,O
24	869	1-Hexanol	111-27-3	alcohols	1.93E-01	1.99E-01	1.13E-01	1.75E-01	1.99E-01	2.69E-01	grassy, marzipan-like	MS, RI
25	877	1-Butanol 3-methyl acetate	123-92-2	esters	6.38E-02	5.83E-02	3.79E-02	2.71E-02	3.98E-02	5.70E-02	banana-like, fruity	MS, RI
26	880	1-Butanol 2-methyl acetate	624-41-9	esters	9.55E-02	6.42E-02	4.01E-02	2.68E-02	4.60E-02	6.82E-02	sweet, fruity	MS, RI
27	890	2-Heptanone	110-43-0	ketones	Nd	1.48E-01	1.03E-01	2.93E-01	6.14E-01	3.18E+00	fruity, soapy	MS, RI
28	899	(*Z*)-4-Heptenal	6728-31-0	aldehydes	Nd	1.94E-02	9.82E-03	1.64E-02	3.56E-02	5.13E-02	fish oil-like	MS, RI,O
29	901	Heptanal	111-71-7	aldehydes	1.08E-02	6.45E-02	8.91E-02	9.20E-02	Nd	Nd	citrus-like, fatty	MS, RI
30	913	(*Z*)-2-Penten-1-ol acetate	42125-10-0	esters	7.70E-02	3.07E-01	2.05E-01	1.79E-01	2.80E-01	3.34E-01	fruity	MS, RI
31	914	Acetic acid pentyl ester	628-63-7	esters	9.38E-02	1.35E-01	2.38E-01	1.95E-01	1.94E-01	7.53E-02	fruity	MS, RI,O
32	920	(*Z*)-3-Hexen-1-ol formate	33467-73-1	esters	4.89E-02	5.71E-02	6.89E-02	4.53E-02	4.24E-02	3.75E-02	——	MS, RI
33	923	2-Methylbut-2-en-1-yl acetate	33425-30-8	esters	1.01E+00	1.57E-01	1.17E-01	8.09E-02	1.37E-01	7.90E-01	——	MS, RI

34	933	(*Z*)-3-Hexenoic acid methyl ester	13894-62-7	esters	1.19E-01	1.54E-01	1.67E-01	1.62E-01	1.80E-01	1.12E-01	——	MS, RI
35	957	(*E*)-2-Heptenal	18829-55-5	aldehydes	8.97E-02	1.31E-01	1.17E-01	2.12E-01	2.59E-01	3.73E-01	rubble	MS, RI,O
36	962	Benzaldehyde	100-52-7	aldehydes	1.53E+01	2.09E+01	1.97E+01	3.44E+01	3.83E+01	6.06E+01	bitter almond-like,	MS, RI
37	971	1-Heptanol	111-70-6	alcohols	2.71E-02	2.00E-02	2.80E-02	2.98E-02	3.07E-02	Nd	fruity, soapy	MS, RI
38	979	1-Octen-3-one	4312-99-6	ketones	1.07E-01	1.31E-01	1.32E-01	2.34E-01	2.98E-01	5.53E-01	mushroom-like	MS, RI
39	980	1-Octen-3-ol	3391-86-4	alcohols	1.63E-01	2.31E-01	2.37E-01	4.75E-01	7.44E-01	1.95E+00	mushroom-like	MS, RI,O
40	988	6-methyl-5-Hepten-2-one	110-93-0	ketones	2.81E+01	2.68E+01	2.60E+01	2.60E+01	3.02E+01	3.88E+01	——	MS, RI
41	991	*β*-Myrcene	123-35-3	alkenes	3.56E+00	3.71E+00	4.61E+00	4.99E+00	5.61E+00	6.74E+00	citrus	MS, RI,O
42	1003	Octanal	124-13-0	aldehydes	8.01E-02	8.23E-02	7.33E-02	7.04E-02	5.89E-02	1.70E-01	citrus	MS, RI,O
43	1008	(*Z*)-3-Hexen-1-yl acetate	3681-71-8	esters	4.74E+01	5.69E+01	6.53E+01	1.63E+01	3.51E+01	1.62E+01	fruity	MS, RI,O
44	1013	Acetic acid hexyl ester	142-92-7	esters	1.66E-01	2.24E-01	1.95E-01	1.28E-01	1.26E-01	1.19E-01	fruity, pear-like	MS, RI
45	1018	*α*-Terpinene	99-86-5	alkenes	1.00E-01	1.43E-01	1.47E-01	2.00E-01	2.53E-01	2.83E-01	terpene-like	MS, RI
46	1022	4-Hexen-1-ol acetate	72237-36-6	esters	Nd	5.08E-02	5.58E-02	5.26E-02	9.35E-02	7.51E-02	——	MS, RI
47	1026	*β*-Cymene	535-77-3	alkenes	1.21E-01	1.60E-01	1.53E-01	3.14E-01	4.09E-01	7.20E-01	——	MS, RI
48	1030	D-Limonene	5989-27-5	alkenes	4.84E-01	7.33E-01	7.69E-01	1.02E+00	1.34E+00	1.63E+00	citrus-like	MS, RI
49	1044	Benzyl alcohol	100-51-6	alcohols	7.00E+01	7.71E+01	7.88E+01	9.31E+01	9.61E+01	1.02E+02	bitter almond-like,	MS, RI
50	1047	Benzeneacetaldehyde	122-78-1	aldehydes	Nd	Nd	Nd	Nd	Nd	1.18E+00	rose,honey	MS, RI,O
51	1049	*β*-Ocimene	13877-91-3	alkenes	1.39E+00	1.55E+00	1.74E+00	1.82E+00	2.69E+00	2.42E+00	citrus	MS, RI,O
52	1055	2,6-Dimethyl-5-heptenal	106-72-9	aldehydes	4.42E-01	5.28E-01	4.97E-01	2.55E-01	1.65E-01	7.61E-02	——	MS, RI
53	1061	*γ*-Terpinene	99-85-4	alkenes	4.79E-02	1.16E-01	1.07E-01	1.47E-01	2.07E-01	2.15E-01	petrol-like	MS, RI
54	1072	Acetophenone	98-86-2	ketones	2.36E-01	2.56E-01	1.95E-01	2.29E+00	4.11E-01	7.40E-01	foxy, bitter almond-like,	MS, RI
55	1076	(*Z*)-Linalool oxide (furanoid)	5989-33-3	alcohols	1.56E+00	2.02E+00	1.11E+00	2.95E+00	3.50E+00	6.91E+00	citrus, floral	MS, RI,O
56	1084	Formic acid phenylmethyl ester	104-57-4	esters	2.11E-01	2.87E-01	3.66E-01	2.73E-01	3.27E-01	3.36E-01	fruity	MS, RI,O
57	1087	3-methyl-Phenol	108-39-4	phenols	Nd	Nd	Nd	Nd	8.71E-02	1.32E-01	smoky, phenolic	MS, RI
58	1091	*α*-Terpinolene	586-62-9	alkenes	1.68E-01	2.85E-01	4.74E-01	6.76E-01	8.45E-01	Nd	——	MS, RI
59	1095	(*E*)-Linalool oxide (furanoid)	34995-77-2	alcohols	3.39E+00	4.38E+00	3.69E+00	5.63E+00	5.83E+00	8.49E+00	citrus	MS, RI,O
60	1102	Benzoic acid methyl ester	93-58-3	esters	3.88E+02	4.04E+02	3.81E+02	3.06E+02	3.61E+02	1.37E+02	fruity, sweet	MS, RI,O
61	1115	Linalool	78-70-6	alcohols	3.65E+02	4.41E+02	5.48E+02	4.87E+02	5.10E+02	3.26E+02	citrus	MS, RI,O
62	1116	6-Methyl-3,5-heptadiene-2-one	1604-28-0	ketones	1.40E+00	1.33E+00	9.28E-01	9.58E-01	1.18E+00	1.45E+00	——	MS, RI
63	1123	Phenylethyl Alcohol	60-12-8	alcohols	9.22E-01	8.09E-01	1.28E+00	9.67E-01	2.09E+00	1.81E+00	rose, honey	MS, RI,O
64	1132	(*E*, *Z*)-2,6-dimethyl-2,4,6-Octatriene	7216-56-0	alkenes	1.15E-01	1.26E-01	1.42E-01	1.46E-01	2.46E-01	2.48E-01	——	MS, RI
65	1134	*E*, *E*-2,6-Dimethyl-1,3,5,7-octatetraene	460-01-5	alkenes	7.01E-02	7.40E-02	9.52E-02	8.86E-02	1.24E-01	1.46E-01	citrus	MS, RI,O
66	1177	Acetic acid phenylmethyl ester	140-11-4	esters	9.31E+02	8.68E+02	1.06E+03	4.44E+02	5.09E+02	2.40E+02	sweet, fruity	MS, RI
67	1181	Benzoic acid ethyl ester	93-89-0	esters	2.51E+00	4.00E+00	1.54E+00	2.21E+01	1.73E+01	5.98E+00	fruity	MS, RI,O
68	1185	(*E*)-Linalool oxide (pyranoid)	39028-58-5	alcohols	3.16E-01	6.14E-01	6.11E-01	7.94E-01	Nd	Nd	earthy	MS, RI,O
69	1188	Benzeneacetic acid methyl ester	101-41-7	esters	1.47E-01	3.87E-01	1.91E-01	1.43E-01	2.11E-01	1.35E+00	floral, fruity	MS, RI,O
70	1187	Terpinen-4-ol	562-74-3	alcohols	Nd	Nd	Nd	2.37E-01	2.49E-01	6.31E-01	earthy, moldy	MS, RI
71	1191	(*E*)-Butanoic acid 3-hexenyl ester	53398-84-8	esters	2.61E+00	3.37E+00	3.21E+01	1.56E+00	1.45E+00	8.06E-01	fruity	MS, RI
72	1202	*α*-Terpineol	98-55-5	alcohols	5.97E-01	Nd	Nd	Nd	Nd	Nd	floral, citrus-like	MS, RI
73	1208	Methyl salicylate	119-36-8	esters	1.94E+02	2.61E+02	3.54E+02	2.83E+02	2.66E+02	1.60E+02	mint-like	MS, RI,O
74	1230	*β*-Cyclocitral	432-25-7	aldehydes	8.26E-02	9.35E-02	1.30E-01	1.72E-01	9.87E-02	Nd	——	MS, RI

75	1232	Nerol	106-25-2	alcohols	2.36E-01	3.09E-01	3.44E-01	4.61E-01	4.87E-01	Nd	rose-like, floral	MS, RI
76	1238	cis-3-Hexenyl-α-methylbutyrate	53398-85-9	esters	1.00E+00	1.10E+00	1.24E+00	9.38E-01	6.58E+00	4.80E-01	——	MS, RI
77	1234	cis-3-Hexenyl isovalerate	35154-45-1	esters	1.75E+00	1.27E+00	8.18E-01	4.01E-01	4.09E-01	5.24E-01	——	MS, RI
78	1257	Geraniol	106-24-1	alcohols	5.68E+00	5.72E+00	8.85E+00	5.74E+00	5.60E+00	3.51E+00	citrus	MS, RI,O
79	1262	Acetic acid 2-phenylethyl ester	103-45-7	esters	3.38E+00	2.26E+00	3.73E+00	1.33E+00	4.25E+00	1.55E+00	honey, floral	MS, RI,O
80	1277	Benzoic acid 2-hydroxy ethyl ester	118-61-6	esters	2.55E+00	4.42E+00	6.47E+00	6.33E+00	4.31E+00	3.69E+00	——	MS, RI
81	1271	Nonanoic acid	112-05-0	others	8.80E-01	5.42E-01	5.51E-01	1.33E+00	6.57E+00	3.65E+00	moldy, pungent	MS, RI
82	1306	Indole	120-72-9	nitrogen heterocycle	2.67E+02	2.77E+02	3.96E+02	1.18E+02	6.31E+01	1.55E+01	camphor	MS, RI,O
83	1320	1-methyl-Naphthalene	90-12-0	others	Nd	1.27E-02	1.65E-02	1.75E-02	2.78E-02	1.75E-02	——	MS, RI
84	1334	3-Phenyl-2-propen-1-ol	104-54-1	alcohols	1.18E+01	1.04E+01	1.42E+01	1.89E+01	1.12E+01	3.54E+00	floral	MS, RI
85	1356	Methyl anthranilate	134-20-3	esters	2.09E+02	2.12E+02	2.87E+02	1.58E+02	1.55E+02	1.29E+02	grape, sweet	MS, RI,O
86	1359	α-Cubebene	17699-14-8	alkenes	1.81E+00	2.37E+00	3.61E+00	2.32E+00	1.22E+00	5.48E-01	——	MS, RI
87	1364	Eugenol	97-53-0	phenols	2.73E+00	2.52E+00	3.46E+00	1.98E+00	1.55E+00	1.41E+00	clove-like	MS, RI
88	1379	Butyl benzoate	136-60-7	esters	2.78E+00	2.83E+00	2.67E+00	2.18E+00	1.88E+00	1.02E+00	——	MS, RI
89	1385	Geranyl acetate	105-87-3	esters	2.87E+00	3.25E+00	4.45E+00	1.54E+00	1.48E+00	4.41E-01	floral	MS, RI,O
90	1387	cis-3-Hexenyl cis-3-hexenoate	61444-38-0	esters	1.34E+00	1.32E+00	2.37E+00	8.99E-01	7.14E-01	2.10E-01	——	MS, RI
91	1393	Butanoic acid 2-methyl phenylmethyl ester	56423-40-6	esters	5.35E-01	7.55E-01	9.15E-01	7.25E-01	6.81E-01	6.24E-01	grape	MS, RI,O
92	1398	Butanoic acid 3-methyl phenylmethyl ester	103-38-8	esters	1.23E+01	6.40E+00	9.74E+00	1.33E+01	1.14E+01	5.60E+00	——	MS, RI
93	1408	cis-Jasmone	488-10-8	ketones	1.39E-01	3.07E-01	3.35E-01	3.20E-01	3.00E-01	2.11E-01	——	MS, RI
94	1419	Dimethyl anthranilate	85-91-6	esters	4.10E+00	5.10E+00	9.29E+00	4.60E+00	4.96E+00	5.44E+00	sweet, floral	MS, RI
95	1425	Ethyl anthranilate	87-25-2	esters	1.13E-01	1.10E-01	1.29E-01	8.18E-02	7.03E-02	1.15E-01	——	MS, RI
96	1436	1,6-dimethyl-Naphthalene	575-43-9	others	1.18E-02	1.67E-02	1.31E-02	1.35E-02	2.30E-02	3.04E-02	——	MS, RI
97	1438	α-Ionone	127-41-3	ketones	5.39E-02	8.32E-02	1.98E-01	1.39E-01	1.36E-01	1.28E-01	floral, violet-like	MS, RI,O
98	1446	Isoamyl benzoate	94-46-2	esters	3.16E-01	3.15E-01	3.95E-01	2.75E-01	1.82E-01	1.52E-01	——	MS, RI
99	1452	Acetic acid cinnamyl ester	103-54-8	esters	1.70E-01	3.29E-01	5.68E-01	1.52E-01	1.41E-01	5.37E-02	floral, rose-like	MS, RI
100	1456	trans-Geranylacetone	3796-70-1	ketones	1.32E+00	2.15E+00	1.42E+00	1.38E+00	1.16E+00	6.60E-01	floral	MS, RI,O
101	1462	Dimethyl phthalate	131-11-3	esters	1.27E+01	3.51E+00	5.64E+00	2.78E+01	1.75E+01	1.33E+00	——	MS, RI
102	1461	γ-Muurolene	30021-74-0	alkenes	2.94E-01	3.24E-01	6.87E-01	2.15E-01	5.13E-02	4.12E-02	——	MS, RI
103	1483	Benzoic acid pentyl ester	2049-96-9	esters	3.56E-01	3.90E-01	5.72E-01	4.05E-01	4.08E-01	1.87E-01	fruity, sweet	MS, RI,O
104	1483	δ-Decenolactone	54814-64-1	esters	8.56E+00	1.13E+01	8.02E+00	1.65E+01	2.44E+00	2.21E+00	sweet, peach-like	MS, RI
105	1547	Dihydroactinidiolide	17092-92-1	esters	8.16E+01	4.81E+01	3.58E+01	1.21E+02	1.19E+02	5.62E+01	——	MS, RI
106	1492	2-Penten-1-ol, benzoate	65416-27-5	esters	7.59E-01	8.07E-01	7.60E-01	6.95E-01	4.25E-01	3.08E-01	——	MS, RI
107	1495	trans-β-Ionone	79-77-6	ketones	8.93E-01	1.26E+00	1.75E+00	1.42E+00	1.40E+00	8.25E-01	floral, violet-like	MS, RI,O
108	1505	Benzyl tiglate	37526-88-8	esters	1.68E+00	2.14E+00	2.69E+00	1.82E+00	1.61E+00	1.57E+00	fruity	MS, RI,O
109	1511	α-Muurolene	10208-80-7	alkenes	4.03E-01	4.98E-01	6.53E-01	4.18E-01	1.81E-01	1.04E-01	——	MS, RI
110	1518	2,4-Di-tert-butylphenol	96-76-4	phenols	4.43E-01	4.52E-01	6.28E-01	6.98E-01	1.05E+00	4.38E-01	phenolic-like,	MS, RI
111	1547	Cubenene	29837-12-5	alkenes	6.79E-01	7.79E-01	1.35E+00	6.92E-01	3.27E-01	6.99E-02	——	MS, RI
112	1553	Hexanoic acid phenylmethyl ester	6938-45-0	esters	1.46E-01	1.99E-01	2.75E-01	2.16E-01	1.95E-01	2.03E-01	——	MS, RI
113	1554	α-Calacorene	21391-99-1	alkenes	3.01E+00	2.80E+00	5.42E+00	2.07E+00	6.31E-01	3.24E-01	——	MS, RI

114	1586	(*Z*)-3-Hexen-1-ol benzoate	25152-85-6	esters	3.08E+02	3.32E+02	4.75E+02	3.44E+02	2.03E+02	1.33E+02	——	MS, RI
115	1593	E-2-Hexenyl benzoate	76841-70-8	esters	3.66E+00	3.30E+00	5.50E+00	4.15E+00	2.24E+00	1.16E+00	——	MS, RI
116	1605	2,2,4-Trimethyl-1,3-pentanediol diisobutyrate	6846-50-0	esters	2.62E-01	2.53E-01	2.88E-01	3.40E-01	4.38E-01	2.32E-01	——	MS, RI
117	1608	Methyl N-acetylanthranilate	2719-08-6	esters	1.00E+02	8.76E+01	7.81E+01	1.24E+02	9.85E+01	4.87E+01	——	MS, RI
118	1617	Cedrol	77-53-2	alcohols	1.10E-01	1.29E-01	2.00E-01	2.31E-01	1.24E-01	1.08E-01	——	MS, RI
119	1658	Methyl jasmonate	1211-29-6	esters	Nd	3.15E+01	3.57E+01	5.94E+01	2.64E+01	8.15E+00	——	MS, RI
120	1654	.tau.-Cadinol	5937-11-1	alcohols	2.66E-01	6.88E+00	8.84E+00	9.39E+00	4.79E+00	3.54E+00	——	MS, RI
121	1668	α-Cadinol	481-34-5	alcohols	2.81E+00	4.08E+00	4.36E+00	5.02E+00	2.12E+00	1.60E+00	——	MS, RI
122	1677	cis-3-Hexenyl salicylate	65405-77-8	esters	2.02E-01	2.83E-01	4.92E-01	1.71E+00	1.49E-01	1.13E-01	——	MS, RI
123	1782	Benzyl Benzoate	120-51-4	esters	1.65E+01	1.81E+01	2.06E+01	1.59E+01	8.45E+00	8.17E+00	——	MS, RI
124	1818	2-Ethylhexyl salicylate	118-60-5	esters	2.38E-01	1.80E-01	2.66E-01	2.66E-01	1.59E-01	1.26E-02	——	MS, RI
125	1890	Benzoic acid 2-hydroxy phenylmethyl ester	118-58-1	esters	6.68E-01	8.04E-01	1.03E+00	6.30E-01	2.44E-01	2.72E-01	——	MS, RI
126	1906	Homosalate	118-56-9	esters	3.09E-01	2.54E-01	2.46E-01	2.39E-01	1.41E-01	Nd	——	MS, RI
127	1994	Hexadecanoic acid ethyl ester	628-97-7	esters	4.45E-02	5.68E-02	1.02E-01	1.11E-01	1.24E-01	1.84E-01	——	MS, RI

Retention index (RI), calculated from the retention times of the compounds and a homologous series of n-alkanes (C6-C25) separated separately by the HP-5MS capillary columns. Nd, the compound was not detected in the samples. Methods of identification: MS, volatiles were identified by mass spectra; RI, retention indices; O, olfactometry.

Table S2

Correlation analysis between the concentration of 9 key aroma compounds and year of storage.

Compounds	2022	2021	2020	2019	2018	2011	r
(*Z*)-4-Heptenal	0	1.3	0.2	1.0	3.3	3.6	0.86
(*Z*)-3-Hexen-1-yl acetate	103.6	122	138.4	40.3	86.1	42.7	-0.67
Benzoic acid methyl ester	577.3	600.7	568	466	538.9	212.8	-0.78
Methyl salicylate	251.9	341.3	465.3	376.2	346.8	206.2	-0.18
Methyl anthranilate	236.8	240	333.6	176.8	170.1	136.8	-0.66
trans-β-Ionone	10.6	10.8	11	10.8	10.8	10.6	-0.07
Linalool	157.7	207.5	278.9	243.1	253.2	132.4	-0.02
Benzoic acid ethyl ester	20.5	32.2	12.9	174.6	136.5	47.8	0.48
Geraniol	5.2	5.2	8	5.3	5.1	3.4	-0.43

r: correlation coefficient value, obtained by correlating the two attributes storage year and concentration using the Correl function.

Table S3

Standard curves of the concentrations of 9 key aroma compounds.

Compounds	CAS	Standard curves	R^2
(*Z*)-4-Heptenal	6728-31-0	y = 0.008451 x + 0.008044	0.996
(*Z*)-3-Hexen-1-yl acetate	3681-71-8	y = 0.5136 x - 5.750	0.991
Benzoic acid methyl ester	93-58-3	y = 0.6869x - 8.7610	0.998
Methyl salicylate	119-36-8	y = 0.7542 x + 4.2272	0.999
Methyl anthranilate	134-20-3	y = 0.8104 x + 17.7562	0.997
trans-*β*-Ionone	79-77-6	y = 2.5170 x - 25.8178	0.995
(*E*)-Linalool oxide (furanoid)	34995-77-2	y = 0.06492 x + 0.3540	0.997
Linalool	78-70-6	y = 1.5245 x + 124.6750	0.995
Benzoic acid ethyl ester	93-89-0	y = 0.12730 x - 0.09957	0.997
(*E*)-Linalool oxide (pyranoid)	39028-58-5	y = 0.01619 x + 0.2004	0.992
Geraniol	106-24-1	y= 1.1722 x - 0.4110	0.990

Table S4

Reference odourants addition to the six tea infusions.

Odourants	Concentration(μg/L)											
	2022		2021		2020		2019		2018		2011	
	infusion	addition	infusion	addition	infusion	addition	infusion	addition	infusion	addition	infusion	addition
(*Z*)-4-Heptenal	0.0	3.6	1.3	2.3	0.2	3.4	1.0	2.6	3.3	0.3	3.6	0.0
(*Z*)-3-Hexen-1-yl acetate	103.6	34.8	122.0	16.4	138.4	0.0	40.3	98.1	86.1	52.3	42.7	95.7
Benzoic acid methyl ester	577.3	23.4	600.7	0.0	568.0	32.7	466.0	134.7	538.9	61.8	212.8	387.9
Methyl salicylate	251.9	213.4	341.3	124.0	465.3	0.0	376.2	89.1	346.8	118.5	206.2	259.1
Methyl anthranilate	236.8	96.8	240.0	93.6	333.6	0.0	176.8	156.8	170.1	163.5	136.8	196.8
trans-β-Ionone	10.6	0.4	10.8	0.2	11.0	0.0	10.8	0.2	10.8	0.2	10.6	0.4
Linalool	157.7	121.2	207.5	71.4	278.9	0.0	243.1	35.8	253.2	25.7	132.4	146.5
Benzoic acid ethyl ester	20.5	154.1	32.2	142.4	12.9	161.7	174.6	0.0	136.5	38.1	47.8	126.8
Geraniol	5.2	2.8	5.2	2.8	8.0	0.0	5.3	2.7	5.1	2.9	3.4	4.6

Odourants, 9 key aroma compounds were screened for addition to the tea infusions

infusion, concentration detected in tea infusions.

addition, concentration of adding to the tea infusion
